# Absorption and Metabolism Characteristics of Rutin in Caco-2 Cells

**DOI:** 10.1155/2013/382350

**Published:** 2013-10-02

**Authors:** Xiaofang Zhang, Jinhui Song, Xiaopeng Shi, Shan Miao, Yan Li, Aidong Wen

**Affiliations:** ^1^Department of Pharmacy, Xijing Hospital, Fourth Military Medical University, Xi'an, Shaan'xi 710032, China; ^2^Department of Otolaryngology, Shenyang 463 Hospital, Xiao He Yan Road 46, Shenyang, Liaoning 110042, China; ^3^School of Pharmacy, Institute of Materia Medica, Fourth Military Medical University, Xi'an, Shaan'xi 710032, China

## Abstract

The intestinal absorption and metabolism characteristics of the potentially beneficial polyphenol rutin were studied by measuring the intracellular accumulation and transport of rutin into Caco-2 cells with the sensitive and reliable analytical method of HPLC-coupled tandem mass spectrometry. Rutin and glucuronidated rutin were absorbed differently by the basolateral and apical membranes, and rutin showed differential permeability through the apical and basolateral sides. Approximately 33% of the rutin was metabolized to glucuronidated rutin, and the intracellular concentration of glucuronidated rutin was much lower than that of parent rutin. P-glycoprotein and multidrug-resistant proteins 2 and 3 were involved in the transmembrane transport and intracellular accumulation of rutin by Caco-2 cells. These results suggest that a specific transport system mediates rutin movement across the apical membrane in Caco-2 cells and that metabolic enzymes are important for this process.

## 1. Introduction

Polyphenols are found in many plants and are abundant in fruits and vegetables [1, 2]. These compounds have attracted much interest owing to clear evidence of their preventative effect in cardiovascular disease and cancer [3, 4]. Rutin, a polyphenol, has received considerable attention as a potential protector against a variety of human diseases. Recent studies have summarized the mechanisms of rutin bioactivity [5–7].

Although rutin has received much attention owing to its multiple biological effects, the limited understanding of its absorption characteristics and metabolic pathways makes it difficult to understand the reasons for its poor bioavailability. In previous reports, intestinal metabolic enzymes, transporters, and even microflora were reported to be the main reasons for the poor bioavailability of natural polyphenols [8, 9].

Intestinal transport proteins, such as P-glycoprotein (P-gp), multidrug-resistant proteins (MRPs), and sodium glucose cotransporters, play key roles in drug transport and accumulation in human intestinal cells [10–12]. P-gp is highly expressed in the cell membrane, belongs to the ATP-binding cassette (ABC) transporter superfamily, and has two nucleotide-binding domains and two halves that each are highly similar to other proteins; each half contains a transmembrane domain [13]. Naturally derived drugs make up a high proportion of P-gp substrates [14]. Although MRPs also belong to the ABC transporter superfamily, they have different substrate specificities than P-gp and can induce multidrug resistance [15]. These transporters are constitutively expressed and abundant in the apical membrane of many epithelial and endothelial barriers [16].

Recently, the human colon adenocarcinoma cell line Caco-2 was used to investigate and evaluate the role of the intestine in drug absorption, transport, and even metabolism [17, 18]. When cultured in an appropriate medium, this cell line can spontaneously differentiate into polarized cells having many enterocyte-like properties of transport-related epithelia [19]. Various transporters such as P-gp and MRPs, which clearly participate in the intestinal transport mechanism and absorption characteristics of certain compounds, are expressed in Caco-2 cells [20, 21]. In addition, cytochrome P450 and UDP-glucuronosyltransferase are expressed in Caco-2 cells [22], and thus this model has been widely used to study the characteristics of drug metabolism in the intestine. 

In addition, although many studies have examined the biological effects of rutin, few studies have examined the absorption and metabolism of rutin, in particular, the intestinal characteristics of such processes. Thus, in the current study we used human Caco-2 cells and HPLC-coupled tandem mass spectrometry (MS/MS) to investigate rutin absorption and metabolism. We evaluated the underlying mechanism by analyzing the related cellular transporters and enzymes involved in these processes.

## 2. Materials and Methods

### 2.1. Chemicals

Rutin and emodin of the highest purity available (98%, as determined by HPLC) were purchased from the National Institute for the Control of Pharmaceutical and Biological Products (Beijing, China). Verapamil, MK 571, and cyclosporine were obtained from Sigma (St. Louis, MO, USA). All chemicals and reagents used were of analytical or HPLC grade.

### 2.2. Cell Culture

Caco-2 cells were obtained from the American Type Culture Collection (Manassas, VA, USA) and maintained in plastic culture flasks (Corning Costar, Cambridge, MA, USA). Cells were cultured in Dulbecco's modified Eagle's medium (DMEM), medium supplemented with 1% nonessential amino acids, 1% L-glutamine, 20% fetal bovine serum, 100 U/mL penicillin, and 0.1 mg/mL streptomycin, and were grown in a humidified atmosphere of 5% CO_2_ in air at 37°C [23]. The cells were subcultured when they reached 80% confluency. 

### 2.3. Cellular Uptake Studies

To test the characteristics of intracellular uptake of rutin using a published method [24], Caco-2 cells were seeded at a cell density of 6 × 10^5^ cells·cm^−2^ on six-well plastic plates. Fresh culture medium was added 24 h before the uptake experiments. One hour before the uptake experiments, cells were preincubated at 37°C in incubation medium including modified Hank's balanced salt solution (HBSS; 137 mM NaCl, 5.4 mM KCl, 1.3 mM CaCl_2_, 0.8 mM MgCl_2_, 0.4 mM KH_2_PO_4_, 0.3 mM NaH_2_PO_4_, and 10 mM HEPES/Tris, pH 7.4). After removal of the incubation medium, different concentrations (from 5 to 200 *μ*M) of rutin were added to the medium for different times (from 10 min to 6 h) in the presence or absence of a transporter inhibitor, such as the P-gp inhibitor verapamil, the MRP2-selective inhibitor cyclosporine, or the MRP inhibitor MK 571. To determine the intracellular concentration of rutin at the end of the incubation period, cell lysates were obtained by freeze-thawing the drug-containing cells in liquid nitrogen for three cycles. Samples were analyzed for rutin metabolites after treatment with *β*-glucuronidase [25].

### 2.4. Cellular Transport Studies

To measure rutin transport into Caco-2 cells, the cells were seeded at a density of 1 × 10^5^ cells·cm^−2^ on Millicell Cell Culture Inserts (Millipore, Billerica, MA, USA). Once the transepithelial electrical resistance value reached more than 300 Ω·cm^−2^ (about 19 to 21 days after seeding), the cells were incubated with fresh medium and used for transport experiments [26]. Rutin was added from the apical (AP) or basolateral (BL) side to evaluate the transport characteristics of rutin into the cells in the presence or absence of the different inhibitors mentioned above. Samples were collected and analyzed with the methods described below. 

### 2.5. Sample Preparation and Quantitative Analysis

A sensitive and high-efficiency method was developed to investigate the changes in rutin and its metabolites transported into Caco-2 cells. Briefly, samples from cellular absorption or transport studies (0.2 mL) were extracted three times with 1 mL ethyl acetate with vigorous vortex mixing. After centrifugation (3000 ×g) for 10 min, the supernatants were reconstituted in 50 *μ*L acetonitrile and evaporated to dryness under N_2_. Rutin and its derivatives were quantified with liquid chromatography—MS/MS with the multiple reaction monitoring model [27].

A Quattro Premier system operating with MassLynx MS software, v4.1 (Waters Corporation, Milford, MA, USA) with electrospray ionization MS/MS to generate negative ions [M-H]^−^ was used. Collision-induced dissociation was achieved using argon as the collision gas. A reverse-phase HPLC system (Symmetry C_18_ column, 50 × 2.1 mm, 5 *μ*m. Waters Corp., Milford, MA, USA) was used to separate the compound with an isocratic mobile phase of 0.1% acetic acid/acetonitrile (20 : 80, v/v). The column temperature was 25°C, and the flow rate was 0.2 mL/min. Using the Bradford method with bovine serum albumin as the standard, total cellular protein concentrations were determined to normalize the intracellular drug concentration between experiments.

### 2.6. Data Analysis

Student's *t*-test was used for statistical analysis, and *P* values < 0.05 were considered significant. 

## 3. Results

### 3.1. Method Development

**Figure 1:**
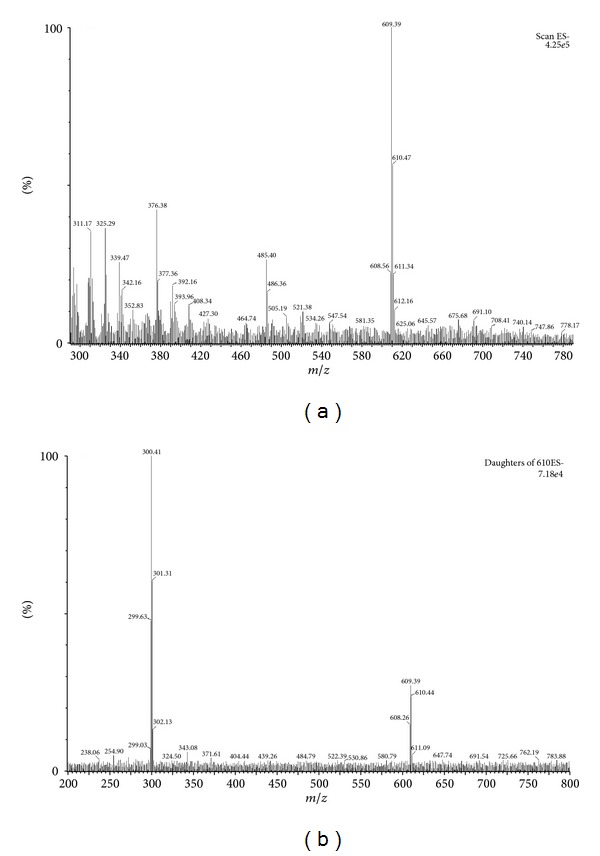
Mass precursor/product ion mass spectra of rutin. The parent ions of rutin were at *m*/*z* 609.4 (a), and the product ions of rutin were obtained at *m/z *300.4 (b). The multiple reaction monitoring mass transition of rutin was at *m*/*z* 609.4→300.4 with a cone voltage of 40 V and collision energy of 32 eV.

**Figure 2:**
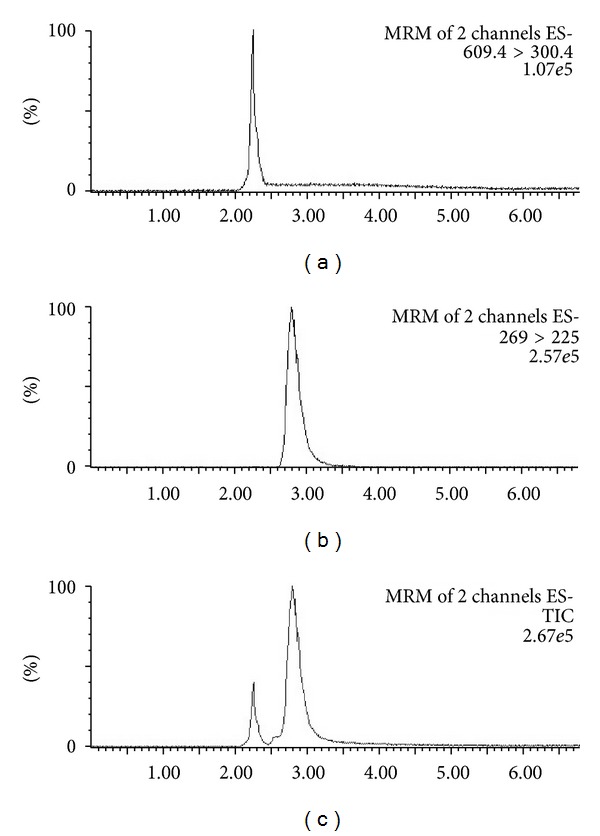
Multiple reaction monitoring chromatograms of rutin and the internal standard. The retention times were 2.25 and 2.85 min for rutin and IS (emodin), respectively, using reverse-phase HPLC with a mobile phase consisting of 0.1% acetic acid/acetonitrile (20 : 80, v/v). (a) Chromatogram for rutin; (b) chromatogram for emodin; (c) chromatogram for both rutin and emodin.

To optimize the detection conditions, a standard rutin solution (1 *μ*g·mL^−1^) was used in the mobile phase. Parent ions and product ions of rutin were selected from spectra after standard solutions were injected into a mass spectrometer in electrospray ionization (ESI) negative-ion mode [M-H]^−^. Using the most sensitive conditions for quantification, the parent ions of rutin were at *m*/*z* 609.4, and the product ions were obtained at *m/z *300.4 (argon was the collision gas). For emodin (IS), the parent and product ions were at *m*/*z* 269 and *m/z *225, respectively. The multiple reaction monitoring mass transition of rutin was at *m*/*z* 609.4→300.4 with a cone voltage of 40 V and collision energy of 32 eV. The transition of IS was at *m*/*z* 269→225 with a cone voltage of 40 V and collision energy of 33 eV (Figure 1). In addition, the chromatographic conditions (Figure 2) were also optimized with retention times of 2.25 and 2.85 min for rutin and IS (emodin), respectively. The specificity of the extraction method and linearity were evaluated in the experiments described below. The regression equation for the standard curve was as follows: *y* = 0.9251*x* − 0.024 (*r* ^2^ ≥ 0.9983, *n* = 6) with the range of 1.0–200.0 ng*·*mL^−1^. The lower limit of quantification (LLOQ), which was defined as the lowest qualification concentration, was 0.5 ng*·*mL^−1^ at a signal-to-noise ratio >10. 

### 3.2. Intracellular Characteristics of Rutin Absorption by Caco-2 Cells

**Figure 3:**
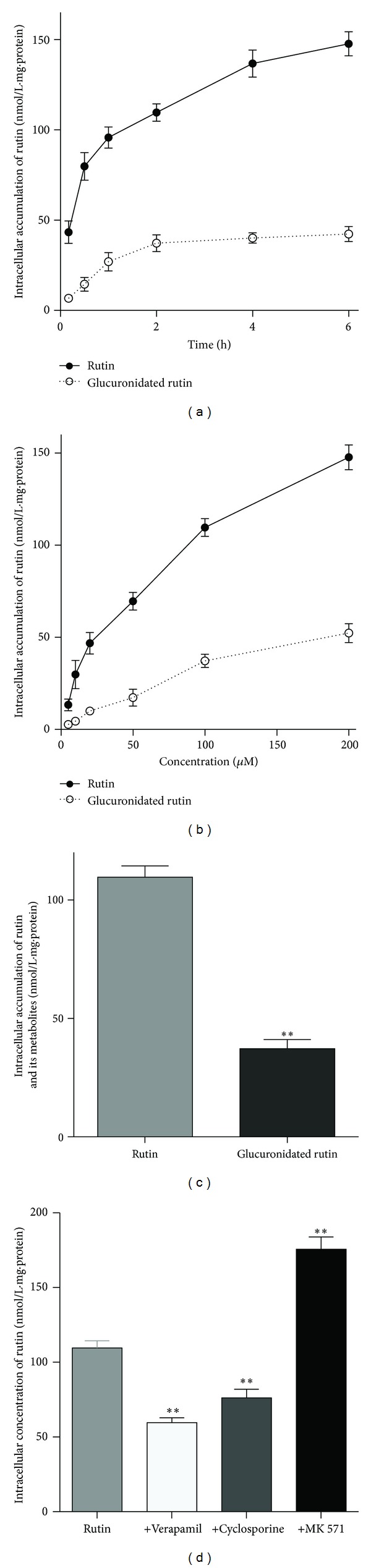
Absorption of rutin in Caco-2 Cells. (a) Time-dependent intracellular accumulation of rutin and its metabolites at 37°C from 10 min to 6 h. (b) Dose-dependent absorption of rutin and its metabolites at 37°C from 5 to 200 *μ*M. (c) Intracellular accumulation of rutin and its metabolites after incubation with 100 *μ*M rutin for 2 h. (d) Intracellular accumulation of rutin in the presence of different inhibitors (10 *μ*M verapamil, 20 *μ*M cyclosporine, 20 *μ*M MK 571, respectively). Each value is the mean ± the SD of seven determinations. Statistical significance: **P < 0.01; *P < 0.05.

Previous reports have shown that polyphenol compounds can be absorbed rapidly by Caco-2 cells [28]. Thus, to investigate the intracellular characteristics of rutin absorption by Caco-2 cells, different concentrations of rutin were incubated with Caco-2 cells for different times (Figure 3). The intracellular accumulation of rutin increased steadily over 6 h (Figure 3(a)). After 2 h of incubation, the concentration had increased about 2.5-fold compared with the first 10 min (43.35 ± 2.25  versus 109.61 ± 4.81 nmol/L·mg*·*protein, *P* < 0.05, *n* = 7). We also examined different concentrations of rutin and observed absorption in a dose-dependent manner from 5 to 200 *μ*M (Figure 3(b)). The intracellular concentration increased more than 10-fold (13.35 ± 3.18  versus 147.73 ± 6.69 nmol/L·mg*·*protein, *P* < 0.01, *n* = 7). In addition, we examined rutin metabolites in Caco-2 cells and detected changes in glucuronidated rutin after enzymatic hydrolysis with *β*-glucuronidase. After 2 h of incubation, the concentration of glucuronidated rutin had increased about 6-fold compared with the first 10 min (6.75 ± 1.73  versus 37.26 ± 4.61 nmol/L·mg*·*protein, *P* < 0.001, *n* = 7), but there is no difference between 2 h and 6 h of incubation (37.26 ± 4.61  versus 42.32 ± 4.13 nmol/L·mg*·*protein, *P* > 0.05, *n* = 7). Approximately 33% of the rutin was metabolized to glucuronidated rutin, and the intracellular concentration of glucuronidated rutin was much lower than that of parent rutin (37.26 ± 3.85  versus 109.61 ± 4.81 nmol/L*·*mg*·*protein, *P* < 0.05, *n* = 7; Figure 3(c)). 

Next, we tested the hypothesis that some drug transporters such as P-gp and MRPs are involved in the intracellular accumulation of rutin in Caco-2 cells. Cells were incubated with rutin for 2 h at 37°C in the presence of a P-gp-selective inhibitor (verapamil) or MRP inhibitors (MK 571 or cyclosporine). After incubation with 100 *μ*M rutin for 2 h, the intracellular concentration of rutin was 109.61 ± 4.81 nmol/L·mg*·*protein in the absence of inhibitors, but interestingly it was clearly lower (109.61 ± 4.81  versus 59.61 ± 3.23 nmol/L·mg*·*protein, *P* < 0.01, *n* = 7) in the presence of 10 *μ*M verapamil (Figure 3(d)). The fact that the intracellular accumulation of rutin was significantly decreased after treatment with a P-glycoprotein inhibitor. Intracellular accumulation of rutin was also lower in the presence of 20 *μ*M cyclosporine, a selective inhibitor of MRP2 (109.61 ± 4.81  versus 76.18 ± 5.83 nmol/L·mg·protein, *P* < 0.05, *n* = 7). Owing to the inhibitory effect of MK 571 on MRP3, which predominantly mediates drug efflux from the basolateral side [29, 30], the intracellular accumulation of rutin was clearly higher in the presence of 20 *μ*M MK 571 (109.61 ± 4.81  versus 175.68 ± 8.19 nmol/L·mg·protein, *P* < 0.05, *n* = 7). These results suggested that the intracellular accumulation of rutin could be observed in a time- and dose-dependent manner and was due to the actions of *β*-glucuronidase and drug transporters. 

### 3.3. Transport Characteristics of Rutin through Caco-2 Cells

**Figure 4:**
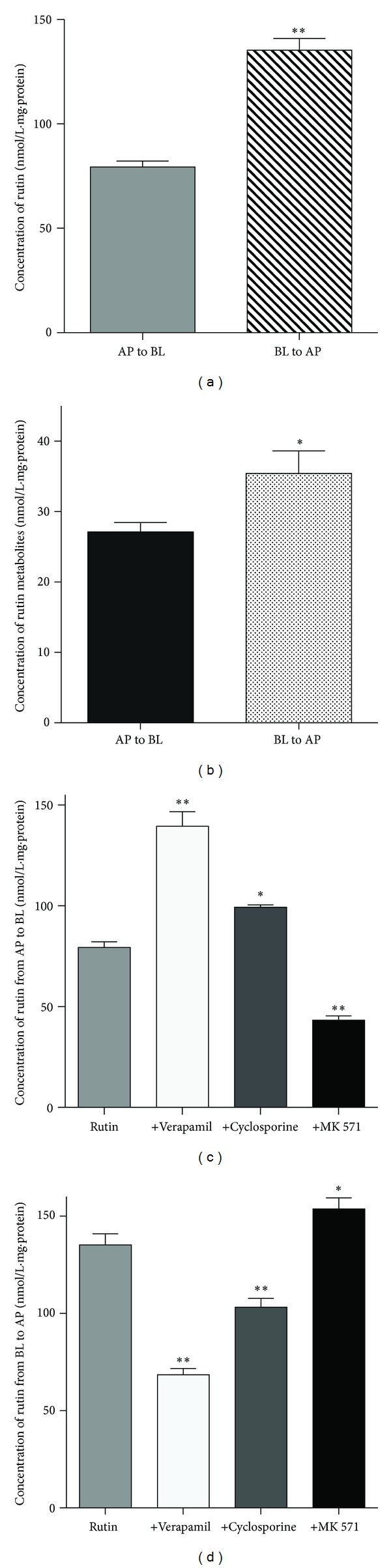
Transport of rutin in Caco-2 Cells. (a) Differences in transport of rutin between the AP to BL direction and the BL to AP direction. (b) Difference in transport of glucuronidated rutin between the AP to BL direction and the BL to AP direction. (c) Transport of rutin in the AP to BL direction in the presence of different inhibitors. (d) Transport of rutin in the BL to AP direction in the presence of different inhibitors. Each value is the mean ± the SD of seven determinations. Statistical significance: **P < 0.01; *P < 0.05.

The transport of rutin and its glucuronidated metabolites into Caco-2 cells was monitored in the apical to basolateral direction and from the basolateral to apical direction in the presence or absence of the above-mentioned inhibitors. Such data would help us understand the underlying mechanism of how the parent rutin and glucuronidated rutin were transported across Caco-2 cells in both directions. First, the permeability values of the parent rutin or glucuronidated rutin were calculated for the different directions (Table 1). For parent rutin (Figure 4(a)), the BL to AP transport was much greater than that from AP to BL (135.23 ± 5.65  versus 79.38 ± 2.79 nmol/L*·*mg*·*protein, *P* < 0.01, *n* = 7). For glucuronidated rutin, we observed different rates of transport in both directions (35.43 ± 3.21 nmol/L*·*mg*·*protein, BL to AP,  versus 27.12 ± 1.34 nmol/L*·*mg*·*protein, AP to BL, *P* < 0.05, *n* = 7), indicating that the permeability value was also dependent on the direction (Figure 4(b)). Verapamil significantly increased the AP to BL transport of rutin (139.45 ± 7.23  versus 79.38 ± 2.79 nmol/L·mg·protein, *P* < 0.01, *n* = 7) but decreased the BL to AP transport (68.46 ± 3.28 versus135.23 ± 5.65 nmol/L·mg·protein, *P* < 0.01, *n* = 7). Cyclosporine also significantly increased the AP to BL transport of rutin (99.36 ± 1.15  versus 79.38 ± 2.79 nmol/L·mg·protein, *P* < 0.05, *n* = 7) but decreased the BL to AP transport (103.23 ± 4.53  versus 135.23 ± 5.65 nmol/L·mg·protein, *P* < 0.01, *n* = 7). After incubation with MK 571, which inhibits MRP3 on the basolateral side, the AP to BL transport of rutin also decreased significantly (43.27 ± 2.18  versus 79.38 ± 2.79 nmol/L·mg·protein, *P* < 0.01, *n* = 7) and increased in the BL to AP direction (153.75 ± 5.66  versus 135.23 ± 5.65 nmol/L·mg·protein, *P* < 0.05, *n* = 7).

## 4. Discussion

To the best of our knowledge, this is the first study to examine the characteristics of absorption, transport, and metabolism of rutin into Caco-2 cells and to also pursue the underlying mechanism. Our novel findings are as follows: (1) intracellular accumulation of rutin was observed in a time- and dose-dependent manner in Caco-2 cells; (2) parent rutin was partly metabolized to glucuronidated rutin in Caco-2 cells; (3) the permeability values of parent rutin were different in the two opposite directions, and the amount of glucuronidated rutin transported was dependent on the direction; (4) P-gp and MRP were involved in the intracellular accumulation and transport by Caco-2 cells. These data suggest that rutin can cross the intestinal epithelium via processes mediated by metabolic enzymes and drug transporters. 

Recent studies have shown that a diet enriched in plant polyphenols correlates with lower incidence of hormone-related cancers and cardiovascular and neurodegenerative diseases. Such a diet also reduces the occurrence of inflammation as well as viral and bacterial infections [31, 32]. Rutin is widely available as a dietary supplement in pharmacies and health food stores. Although much evidence has shown that rutin positively affects human health, the characteristics of its intestinal absorption and metabolism, especially the underlying mechanisms, are still not clear.

Although Caco-2 cells are derived from human colonic adenocarcinoma, the characteristics of expression of P-gp, MRP2, and MRP3 in these cells permit evaluation of the intestinal absorption and transport of natural products and reflect processes that occur in normal intestinal epithelium [30, 33]. Furthermore, Caco-2 cells also express drug-metabolizing enzymes such as glucuronyl or sulfate transferase [34, 35]. Thus, to address the issue of absorption and transport of rutin in the current study, we developed sensitive and reliable analytical methods that were also useful for gaining insight into the mechanism governing these processes in human Caco-2 cells, which are widely accepted as a model of human intestinal absorption. 

P-gp, which transports hydrophobic drugs out of cells in an ATP-dependent manner, is highly expressed in the brain, liver, intestine, kidney, and placental tissues. P-gp transports drugs and xenobiotic compounds ingested with food from the apical side of intestinal epithelial cells, thus modulating the intestinal absorption of xenobiotic compounds [10, 36]. To test the effects of integral membrane transporters on rutin absorption and transport, inhibition experiments were performed to investigate the intracellular accumulation of rutin in the absence or presence of verapamil and cyclosporine, inhibitors of P-gp and various MRPs, respectively. The intracellular accumulation of rutin clearly decreased after pretreatment with verapamil and cyclosporine, indicating that the intracellular accumulation and transport processes of rutin in intestinal epithelial cells are regulated by these transporters. Based on the results, P-gp, which is an efflux transporter, not only exports the parent rutin to the apical side but also sequesters metabolites in the cytoplasm and then pumps them out once the complex merges into the cell membrane. Such an efflux pump protects the sensitive areas of the body from xenotoxins present in our diet.

In addition, MRP2 mediates the cellular export of phase II conjugates from the liver, intestine, and kidneys. Moreover, owing to the high expression of MRP2 in the intestinal tract [37, 38], we hypothesized that MRP2 is involved in the cellular excretion of phase II conjugates of rutin. This hypothesis is supported by the fact that the permeability values of glucuronidated rutin were lower in the AP to the BL direction than in the opposite direction. The data also showed that rutin was extensively metabolized to glucuronidated rutin by phase II enzymes after incubation with Caco-2 cells for 1 h. 

Our results suggest that P-gp and MRP2, which are localized in the apical and basolateral membranes, are capable of exporting rutin, effectively opposing its absorption and intracellular accumulation. It is important that the toxicity of applied rutin decreases because intestinal P-gp and MRP reduce rutin absorption. Drug-drug interactions will unpredictably affect the toxicity of rutin if rutin is coadministered with other P-gp or MRP inhibitors. Information regarding the functional characteristics of drug transporters is important for improving drug design and delivery by considering possible interactions with specific transporter proteins. Our data show that MRP2 and MRP3 differentially modulate the absorption and transport of rutin and its metabolites owing to the differential distribution of these transporters in the apical and basolateral membranes. The balance between different subtypes of MRPs and P-gp may affect the absorption of rutin in Caco-2 cells. Such balance may favor efflux by P-gp and MRP2, or it may shift to favor influx by MRP3.

## 5. Conclusion

Rutin was pumped out of the cells by P-gp and MRP or metabolized by a phase II metabolic enzyme such as glucuronyl transferase in Caco-2 cells. Glucuronyl transferase may play an important role in drug accumulation, transport, and phase II metabolism in the human intestine. Elucidation of the mechanism of intestinal absorption of rutin will assist the development of highly efficient and absorbable drugs based on the original rutin structure. In addition, detailed information about transporter functions may be important for clarifying possible drug-drug interactions. We show that a specific transport system mediates rutin movement across the apical membrane in Caco-2 cells. Thus, strategic administration of intestinal P-gp or a MRP inhibitor may improve the therapeutic effect of orally administered rutin.

## Figures and Tables

**Table 1:** Permeability (*P*_app_) of polyphenol compounds.

Compound	*P*_app_ (cm · s^−1^ · 10^−6^)	
	AP to BL	BL to AP
Rutin	10.32 ± 1.25	17.45 ± 2.53^1^
Glucuronidated rutin	5.21 ± 0.32	7.02 ± 1.31^2^

Mean ± SD; *n* = 6.

^1^Student's *t-*test: significantly different from the AP to BL direction, *P* < 0.01.

^2^Student's *t-*test: significantly different from the AP to BL direction, *P* < 0.05.
